# Experiences with hospital-to-home transitions: perspectives from patients, family members and healthcare professionals. A systematic review and meta-synthesis of qualitative studies

**DOI:** 10.1080/09638288.2024.2384624

**Published:** 2024-08-05

**Authors:** J.W.M. van Grootel, R.J. Collet, J.M. van Dongen, M. van der Leeden, E. Geleijn, R. Ostelo, M. van der Schaaf, S. Wiertsema, M.E. Major

**Affiliations:** a Department of Rehabilitation Medicine, Amsterdam UMC location University of Amsterdam, Amsterdam, The Netherlands; b Ageing and Vitality, Amsterdam Movement Sciences, Amsterdam, The Netherlands; c Musculoskeletal Health, Amsterdam Movement Sciences, Amsterdam, The Netherlands; d Department of Health Sciences, Faculty of Science, Vrije University Amsterdam, Amsterdam, The Netherlands; e Department of Epidemiology and Biostatistics, Amsterdam UMC, Vrije Universiteit Amsterdam, Amsterdam, The Netherlands; f Faculty of Health, Center of Expertise Urban Vitality, Amsterdam University of Applied Sciences, Amsterdam, The Netherlands; g Faculty of Health, Department of Physical Therapy, Amsterdam University of Applied Sciences, Amsterdam, The Netherlands

**Keywords:** Transitional care, healthcare professionals, hospital discharge, care continuity, patients

## Abstract

**Purpose:**

Multiple studies have explored the needs and experiences of patients, family members, and healthcare professionals regarding hospital-to-home transitions. Our study aimed to identify, critically appraise, and summarize these studies in a qualitative meta-synthesis.

**Materials and methods:**

Medline, CINAHL and Embase were systematically searched to identify eligible articles from inception to June 2024. Qualitative studies were included and critically appraised using the Critical Appraisal Skills Program. Insufficient-quality papers were excluded. We performed a meta-synthesis following (1) open coding by two independent researchers and (2) discussing codes during reflexivity meetings.

**Results:**

Ninety-eight studies were appraised, of which 53 were included. We reached thematic saturation, four themes were constructed: (1) care coordination and continuity, (2) communication, (3) patient and family involvement, and (4) individualized support and information exchange. For patients and families, tailored information and support are prerequisites for a seamless transition and an optimal recovery trajectory after hospital discharge. It is imperative that healthcare professionals communicate effectively within and across care settings to ensure multidisciplinary collaboration and care continuity.

**Conclusions:**

This study identifies essential elements of optimal transitional care. These findings could be supportive to researchers and healthcare professionals when (re)designing transitional care interventions to ensure care continuity after hospital discharge.

## IMPLICATIONS FOR REHABILITATION

- Patients and their families need to receive tailored information and support, which are prerequisites for a seamless transition from hospital to home
- Professionals must communicate effectively within and across hospital and primary care settings
- Professional roles should be clarified to ensure effective collaboration and continued high-quality care after hospital discharge.
- Integrated allied health pathways addressing coordination and communication are needed to ensure seamless transitions

## Introduction

Over the past 20 years, global healthcare has changed due to the aging of the population and the increasing prevalence of multimorbidity [1]. In the Netherlands, for example, about 5.7 million people were diagnosed with two or more chronic conditions in 2021 [2]. In the meantime, the average hospital length of stay for each admission has been steadily decreasing from eight to five days in 2022, among countries belonging to the Organization for Economic Cooperation and Development countries, for reasons of curbing healthcare costs [3]. A shorter hospital stay generally results in patients being discharged while still needing follow-up care. Careful coordination between hospital- and primary care providers is therefore needed to ensure continuity of care and optimal recovery. In the past, transitional care interventions have been developed to ensure the coordination and continuity of care across various locations or levels of care [4]. These interventions, which were historically often monodisciplinary in nature, focused mainly on medical care needs, such as medication adherence and were found to reduce hospital readmission rates and improve patient outcomes [5–7]. With the expected increased complexity of patients’ follow-up care needs, such monodisciplinary interventions may no longer be sufficient [8,9].

Ensuring continuity and quality of care for patients with complex care needs requires a more holistic approach to transitional care by a range of healthcare professionals. This means providing not only medical care but also care provided by allied health professionals such as physical therapists, occupational therapists, dieticians, and/or speech and language therapists [8]. The Commonwealth Fund International Health Policy reported that up to 35% of patients in high-income countries encounter difficulties with care coordination after discharge. These issues may arise from situations where professionals responsible for follow-up care are not being provided with patients’ medical histories or receive conflicting information, which may, in turn, delay patient recovery [10,11]. Hence, transitional care interventions are required to provide patients with a seamless hospital-to-home transition and continuity of care by optimizing collaboration between hospital and primary care professionals.

In past years, many qualitative studies have investigated the needs and experiences of patients, family members, and healthcare professionals regarding continuity and coordination of care during hospital-to-home transitions. These studies generally investigated specific patient or professional populations or patients’ experiences with certain aspects of the transition [12,13]. As valuable lessons can be learned from these individual studies regarding optimizing hospital-to-home transitions, conducting a meta-synthesis of qualitative studies on this topic can provide a comprehensive overview and understanding of patient and (allied) health professional needs regarding this sensitive moment in the recovery phase after hospital admission.

Therefore, this study aimed to identify and critically appraise the current body of qualitative evidence investigating patients’, family members’, and (allied health) professionals’ experiences and needs, surrounding care continuity during and after the transition from hospital to home. An overarching synthesis is provided of high-quality study results with recommendations for transitional care interventions.

## Materials and methods

**Figure 1.**
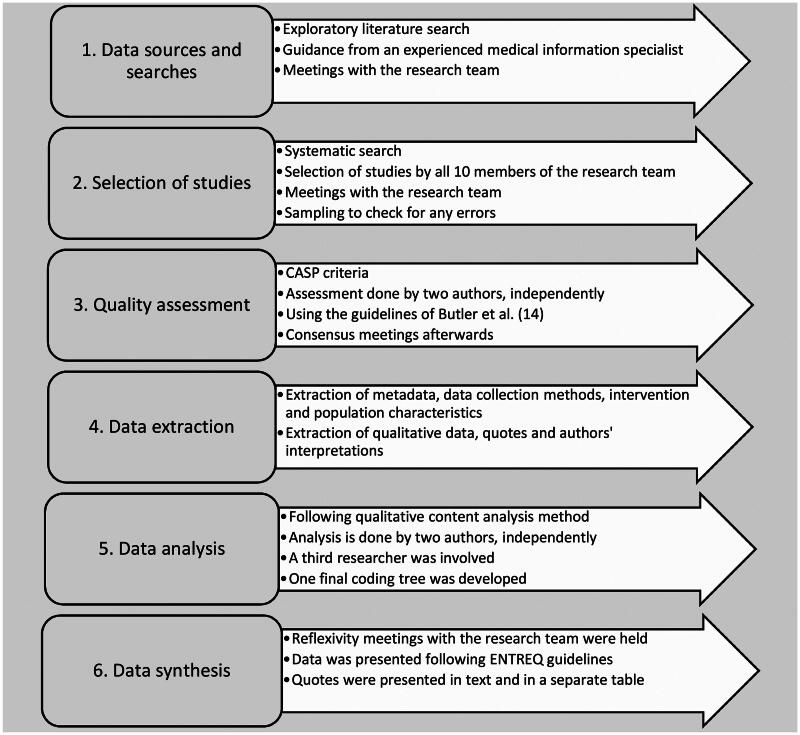
Overview of the steps conducted in this meta-synthesis.

The protocol for this systematic review was registered in Prospero (CRD42023421423). This study adhered to the Preferred Reporting Items for Systematic Reviews and Meta-Analyses (PRISMA) and ENTREQ. Figure 1 presents the design of this meta-synthesis.

### Data sources and searches

Qualitative or mixed-method studies using qualitative data collection methods, such as semi-structured interviews or focus groups, were eligible for review. Electronic searches in Medline, CINAHL and Embase were conducted from inception until June 2024. Additional records were identified through the snowball method using Google Scholar [14]. An experienced medical information specialist conducted the search (Appendix Table A1).

**Table 1.** Quality appraisal.

Firt authors’ last name	Year	CASP items										Total score	Category	Reason for exclusion
		1	2	3	4	5	6	7	8	9	10			
Allen	2020	1.0	1.0	0.5	0.5	1.0	0.5	1.0	0.0	1.0	1.0	7.5	Moderate	
Allen	2018	1.0	1.0	1.0	1.0	1.0	0.0	1.0	0.5	1.0	0.5	7.5	Moderate	
Agerholm	2023	1.0	1.0	0.5	0.5	1.0	1.0	1.0	1.0	0.5	1.0	8	Moderate	
Allen	2022	1.0	1.0	1.0	1.0	1.0	0.5	1.0	0.5	1.0	1.0	9	High	
Allen	2022	1.0	1.0	1.0	0.5	1.0	0.5	1.0	0.5	1.0	1.0	8.5	Moderate	
Antony	2018	1.0	1.0	0.5	0.5	1.0	0.5	1.0	0	1.0	0.5	7	Fair	
Arora	2010	1.0	1.0	0.5	0.5	0.0	1.0	1.0	0.5	0.5	0.0	6	Fair	
Ayele	2019							0.0					Exclude	Nr. 7 = 0.0
Backman	2018	1.0	1.0	1.0	0.5	0.5	0.5	1.0	0.5	1.0	0.0	7	Fair	
Baxter	2020	1.0	1.0	1.0	0.5	1.0	0.5	1.0	0.5	1.0	0.5	8	Moderate	
Brez	2009	1.0	1.0	1.0	1.0	0.5	1.0	1.0	0.0	1.0	0.5	8	Moderate	
Brooke	2018	1.0	1.0	1.0	0.5	1.0	0.0	1.0	0.5	1.0	1.0	8	Moderate	
Crawshaw	2021	1.0	1.0	0.0	1.0	1.0	0.0	1.0	1.0	1.0	0.5	7.5	Moderate	
Clair	2017	0.5	1.0	0.0	1.0	0.5	1.0	1.0	0.0	0.5	0.0	5.5	Exclude	<6.0
Cobley	2013	1.0	1.0	0.5	0.5	1.0	0.0	1.0	1.0	1.0	0.5	7.5	Moderate	
Coleman	2015	1.0	1.0	0.5	0.5	0.5	0.0	1.0	0.0	0.0	0.5	5	Exclude	<6.0
Davis	2012	0.5	1.0	0.5	0.5	1.0	0.5	1.0	0.5	1.0	0.0	6.5	Fair	
de Vos	2017	1.0	1.0	1.0	0.5	0.5	0.0	1.0	0.0	0.0	0.5	5.5	Exclude	<6.0
Devriendt	2013	1.0	0.5	0.0	0.5	0.5	0.0	1.0	0.0	0.5	0.5	4.5	Exclude	<6.0
Domu	2021	1.0	1.0	1.0	0.0	1.0	0.5	1.0	0.0	1.0	0.5	5.5	Exclude	<6.0
Doos	2015	1.0	1.0	1.0	1.0	1.0	0.0	1.0	0.5	0.5	0.5	7.5	Moderate	
Dow, B	2007	1.0	0.5	0.0	1.0	0.5	0.0	1.0	0.0	1.0	0.5	5.5	Exclude	<6.0
Dutton	2014	1.0	1.0	0.5	0.5	0.5	0.0	1.0	0.5	1.0	0.5	6.5	Fair	
Fox	2023	1.0	1.0	0.0	1.0	0.5	0.0	1.0	0.5	1.0	0.5	6.5	Fair	
Gotlib	2018							0.0					Exclude	Nr. 7 = 0.0
Graham	2009							0.0					Exclude	Nr. 7 = 0.0
Greysen	2014	1.0	1.0	0.5	0.5	0.5	0.0	1.0	0.0	1.0	0.0	5.5	Exclude	<6.0
Groene	2012	1.0	1.0	0.0	0.5	1.0	0.0	1.0	1.0	0.5	0.5	6.5	Fair	
Grootel	2024	1.0	1.0	0.5	1.0	1.0	1.0	1.0	1.0	1.0	0.5	9.0	High	
Guassora	2015	0.5	1.0	1.0	0.5	0.0	0.0	1.0	0.5	1.0	0.0	5.5	Exclude	<6.0
Gustafsson	2013	1.0	0.5	1.0	1.0	0.5	0.0	1.0	0.5	1.0	1.0	7.5	Moderate	
Guzman	2022	1.0	1.0	0.0	0.5	0.5	0.0	1.0	0.0	1.0	0.5	5.5	Exclude	<6.0
Hansson	2018	1.0	1.0	0.5	0.5	0.5	0.0	1.0	0.0	0.5	0.5	5.5	Exclude	<6.0
Harvey	2017	1.0	1.0	1.0	1.0	0.5	0.0	1.0	0.5	1.0	0.0	7	Fair	
Humphries	2019	1.0	1.0	0.5	0.5	1.0	1.0	1.0	1.0	1.0	0.5	8.5	Moderate	
Jepma	2021	1.0	1.0	1.0	0.5	1.0	1.0	1.0	1.0	1.0	1.0	9.5	Moderate	
Jones	2015	1.0	1.0	0.5	1.0	0.5	0.5	1.0	1.0	1.0	0.5	8	Moderate	
Kable	2015	1.0	1.0	0.5	1.0	0.5	0.0	1.0	0.0	1.0	0.5	6.5	Fair	
Kangovi	2014	1.0	1.0	0.5	1.0	1.0	0.5	1.0	0.5	1.0	1.0	8.5	Moderate	
Kelly	2016	1.0	1.0	0.5	1.0	1.0	0.0	1.0	1.0	1.0	0.0	7.5	Moderate	
Kimmel	2016	1.0	1.0	0.5	1.0	1.0	1.0	1.0	0.0	1.0	0.5	8	Moderate	
King	2022	1.0	1.0	1.0	0.5	1.0	0.0	1.0	1.0	1.0	1.0	8.5	Moderate	
Kokorelias	2023	1.0	1.0	1.0	1.0	1.0	1.0	1.0	1.0	1.0	1.0	10	High	
Kokorelias	2023	1.0	1.0	0.5	1.0	1.0	0.5	1.0	1.0	1.0	1.0	9	High	
Lilleheie	2020	1.0	1.0	0.5	1.0	1.0	1.0	1.0	1.0	1.0	0.5	9	High	
Lou	2016	1.0	1.0	0.5	0.5	1.0	0.0	1.0	1.0	1.0	1.0	8	Moderate	
Major	2021	1.0	1.0	0.5	1.0	0.0	0.0	1.0	0.0	1.0	0.5	6	Fair	
Major	2019	1.0	1.0	1.0	1.0	1.0	0.0	1.0	1.0	1.0	1.0	9	High	
Maximos	2024	1.0	1.0	1.0	1.0	1.0	0.5	1.0	1.0	1.0	0.5	9	High	
McFadden	2022	1.0	1.0	0.0	1.0	0.5	0.0	1.0	0.0	0.5	1.0	6	Fair	
Muhamad	2022	1.0	1.0	0.5	0.0	0.5	0.0	1.0	0.0	0.5	0.0	4.5	Exclude	<6.0
Naylor	2009	1.0	1.0	0.5	0.5	0.0	0.5	1.0	0.0	0.0	0.0	4.5	Exclude	<6.0
Nissim	2014	1.0	1.0	0.5	0.5	1.0	0.5	1.0	0.0	0.5	0.5	6.5	Fair	
Oravec	2022	1.0	1.0	0.5	1.0	1.0	0.0	1.0	0.5	1.0	1.0	8	Moderate	
O’neill	2024	1.0	1.0	0.0	0.5	0.5	0.0	1.0	0.0	0.5	0.0	4.5	Exclude	<6.0
Park	2022	1.0	1.0	0.5	0.5	1.0	0.0	1.0	0.0	0.5	0.5	6	Fair	
Persson	2022	1.0	1.0	1.0	0.5	1.0	1.0	1.0	1.0	1.0	0.5	9	High	
Petersen	2019	1.0	1.0	1.0	0.0	0.5	0.5	1.0	0.5	0.5	0.5	6.5	Fair	
Prinjha	2009	1.0	1.0	0.5	0.5	0.5	0.0	1.0	0.0	0.5	1.0	6	Fair	
Rupa	2022	1.0	1.0	0.0	0.0	1.0	0.0	1.0	0.5	0.5	0.0	5	Exclude	<6.0
Rustad	2016	1.0	1.0	0.5	1.0	1.0	0.0	1.0	1.0	1.0	0.5	8	Moderate	
Sandlund	2024	1.0	1.0	1.0	1.0	1.0	1.0	1.0	1.0	1.0	0.0	9	High	
Shannon	2022	1.0	1.0	0.5	1.0	1.0	0.5	1.0	0.5	1.0	1.0	8.5	Moderate	
Slager	2017							0.0					Exclude	Nr. 7 = 0.0
Stephens	2013	1.0	1.0	0.0	1.0	0.5	0.0	1.0	0.0	1.0	0.0	5.5	Exclude	<6.0
Strunin	2007	1.0	1.0	0.5	1.0	0.5	0.0	1.0	0.5	1.0	0.5	7	Fair	
Sun	2023	1.0	1.0	0.5	0.5	1.0	0.5	1.0	0.0	1.0	0.5	7	Fair	
Thys	2024	1.0	1.0	0.5	1.0	1.0	0.0	1.0	1.0	1.0	0.5	8	Moderate	
Swinkels	2009	0.5	1.0	1.0	0.5	1.0	0.0	1.0	0.0	1.0	1.0	7	Fair	
Verweij	2021	1.0	1.0	0.5	0.5	1.0	0.0	1.0	0.0	1.0	0.5	6.5	Fair	
Vogel	2024	1.0	1.0	0.0	0.5	0.5	1.0	1.0	0.5	1.0	0.5	7	Fair	
Watts	2005	0.0	1.0	0.5	0.5	0.5	1.0	1.0	0.0	0.5	0.5	5.5	Exclude	<6.0
Witt	2024	1.0	1.0	1.0	1.0	1.0	0.0	1.0	1.0	1.0	0.0	8	Moderate	

*Abbreviations*: CASP: Critical Appraisal Skills Program; Nr: number.

1. Was there a clear statement of the aims of the research?

2. Is a qualitative methodology appropriate?

3. Was the research design appropriate to address the aims of the research?

4. Was the recruitment strategy appropriate to the aims of the research?

5. Was the data collected in a way that addressed the research issue?

6. Has the relationship between researcher and participants been adequately considered?

7. Have ethical issues been taken into consideration?

### Selection of studies

Studies were eligible if they: (1) applied qualitative data collection methods, such as semi-structured interviews and/or focus groups, (2) included adult patients, informal carers, and/or healthcare professionals, and (3) focused on experiences around the hospital-to-home transition and/or experiences with transitional care interventions. Studies investigating experiences with mono-disciplinary transitional care interventions (e.g. telephone calls by a nurse practitioner) were excluded. For patients with complex care needs, transitional care interventions generally require a multidimensional approach leading to the exclusion of studies on single, monodisciplinary care interventions. Also, studies focusing on populations suffering from singular psychological issues or requiring palliative care were excluded, as for these populations, transitional care experiences are expected to be different. Retrieved records were imported into Rayyan (http://rayyan.qcri.org). Title and abstract screening was conducted in two stages: (1) after duplicate removal, titles and abstracts were initially screened (by one reviewer) to filter out articles that did not answer the research question (e.g. studies focusing on medication interventions) and (2) titles and abstracts of the remaining potentially eligible articles were then re-screened by two reviewers independently. Next, full texts were assessed for eligibility by two reviewers (JvG and RC). Disagreements between reviewers were resolved during a consensus meeting.

### Quality assessment

Each article was independently assessed by two researchers (JvG and RC) using the Critical Appraisal Skills Program checklist for qualitative research (CASP) [15,16]. The CASP checklist consists of 10 items assessing the credibility of the evidence. Butler et al. recommended that qualitative systematic reviews rely on well-planned, peer-reviewed protocols for trustworthy and clinically useful results. Articles lacking a statement on ethical approval were excluded (item nr. 7 = 0.0), as were those with a sum score <6.0 [16]. Finally, the papers were categorized into high-quality (score 9.0–10.0), moderate-quality (score 7.5–9.0), and fair-quality (score 6.0–7.5).

### Data extraction

The following data were extracted: author, year, country, aim, patient population (i.e., older, cardiac, stroke patients etc.), occupation of professional interviewees (nurse, allied healthcare professional etc.), care setting of the professional interviewees (primary care, community care, rehabilitation center, hospital etc.), demographics, design and data collection method and qualitative outcomes (quotes and authors’ interpretative analysis).

### Data analysis and synthesis

We used qualitative content analysis to analyze the data and followed the Enhancing Transparency of Reporting the Synthesis of Qualitative Research (ENTREQ) statement [17,18]. JvG and RC thoroughly read all included papers. First-order constructs (direct quotes of participants) and second-order constructs (study authors’ interpretations) relevant to the aim of this meta-synthesis were imported into MAXQDA 2022 (VERBI Software, 2021) [19]. JvG and RC independently coded first, and second-order constructs of two qualitative studies, and codes were compared to ensure consistency in the analysis, after which each author individually coded half of the remaining papers. Throughout the analysis, JvG and RC met after each five studies to discuss new constructed codes. Once the final list of codes was developed by the researchers, the two coders grouped conceptually similar constructs from the different papers to identify categories with a third researcher (MM). To improve the quality and rigour of the study, the results were discussed during several reflexivity meetings involving members of the research team (JvG, RC, MM, SW, JvD, EG, MvdL) [20]. These meetings were pragmatic, gave structure to an inductive method of analysis, and led to the final themes and codes. In these meetings, we discussed when the point of thematic saturation was reached, i.e., when no new themes could be constructed from the set of codes. As a last step, categories were developed to produce third-order constructs (views and interpretations of our research team) expressed as higher-order themes and subthemes. We performed a secondary analysis to provide insight in the distribution of the results per country to assess heterogeneity and external validity of our results (Supplementary File 2).

## Results

**Figure 2.**
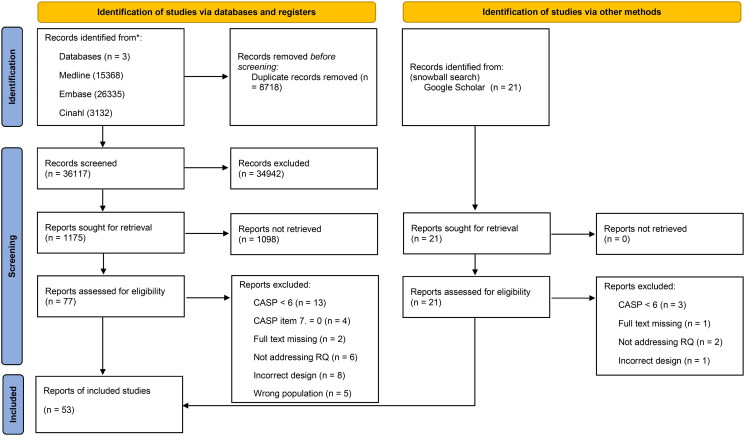
PRISMA Flowchart selection process for qualitative meta synthesis. *Abbreviations*: CASP: critical appraisal skills program checklist for qualitative research.

After duplicate removal, titles and abstracts of the remaining records (*n* = 36,117) were screened for eligibility, leaving 1175 studies meeting the inclusion criteria (Figure 2 PRISMA flowchart). A total of 1098 records were excluded based on screening on full text, 77 records remained. Twenty-one additional papers were identified through a snowball search. Ninety-eight articles were appraised, after which 45 articles were excluded. Reasons for exclusion were CASP < 6.0 or CASP item 7 = 0 (*n* = 20, 44%), not addressing the research question (*n* = 8, 18%), incorrect design and/or population (*n* = 14, 31%), and full-text missing (*n* = 3, 7%). Finally, 53 studies were included of which 39 (74%) investigated experiences, perceptions, or needs regarding hospital-to-home transitions from patients’, family caregivers’, or healthcare professionals’ perspectives, and fourteen studies (26%) investigated (experiences with) specific multidimensional transitional care interventions. Results are presented following the ENTREQ guideline.

### Study and population characteristics

We included studies published between 2007 and 2024 representing populations of fourteen countries, the majority of which were published in the United States (*n* = 10, 19%), the United Kingdom (*n* = 8, 16%), and Australia (*n* = 9, 18%). The following patient populations were included in the studies: older patients (*n* = 23), surgical patients (*n* = 9), cardiac patients (*n* = 3), stroke patients (*n* = 2), diabetic patients (*n* = 2) and other/not specified (*n* = 14). Twenty-nine (55%) studies investigated the perspectives of patients and family caregivers, and sixteen (30%) explored healthcare professionals’ perspectives. Eight studies (15%) explored the combined perspectives of patients, family caregivers, and healthcare professionals. A detailed overview of the study and population characteristics can be found in Supplementary File 1.

### Quality appraisal results

Nine (17%) studies were of high quality [21–28], 24 (45%) of moderate quality [28–55], and 20 (38%) of fair quality [13,56–72] (Table 1). Studies ranked as being of moderate- or fair methodological quality scored lower because they either did not report the relationship between the researcher and the participants, lacked details on the rigor of data analysis, and/or lacked information on the added value of the research was.

### Data synthesis

**Figure 3.**
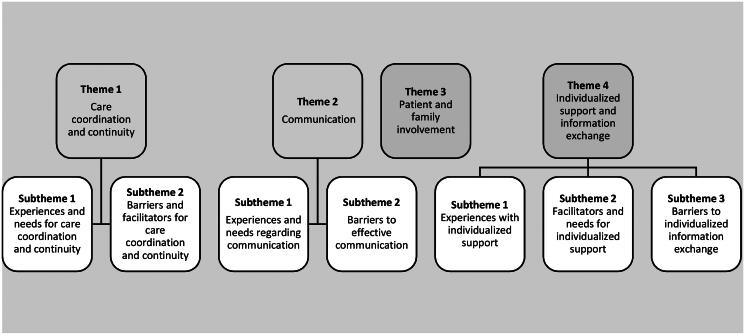
Coding tree: themes and subthemes.

Thematic saturation was reached in this data synthesis after several reflexivity meetings with the research team. At that point, the research team agreed that no new themes were constructed based on the latest articles, and hence that adding more would not reveal new information or insights pertaining to the research question (i.e., additional core concepts related to transitional care experiences). Based on our data synthesis we constructed four themes: (1) care coordination and continuity, (2) communication, (3) patient and family involvement and (4) individualized support and information exchange (Figure 3). Quotes related to themes and subthemes are depicted in Tables 2 and 3.

**Table 2.** Supporting data: quotes related to the first two themes and subthemes.

Theme 1: Care coordination and continuity [13, 21–63]	
Experiences and needs for care coordination and continuity	“How am I supposed to take a 94-year-old lady downtown to (an appointment) park the car, and it’s right near the Byward market, better of taking a taxi, honest to God. And I never took her, I can’t get her out of the house for anything unless you are taking her by, you know, an ambulance, God forbid” [45].
	“Really, not one **** is being done [with the referral letter from the hospital], they open it and just continue with their own program anyway.” [13]
	“Safe transitions of care were perceived not only on knowing the patient well, but also on knowing other team members and those from different teams and settings” [29].
Barriers for care coordination and continuity	“These two sectors [primary care and hospital] operate with distinct care agendas. The practical logic of the primary sector is a holistic approach to care and treatment, whereas the hospital’s agenda is to minimize the length of admission” [22].
	“Multiple individuals in both hospitalist and primary care professional groups described having little time for coordination of care around patient hospitalizations, which compounded the frustration they felt when they had difficulty reaching each other by phone” [35].
Theme 2: Communication [13,15,22,23,25,26,29,31,33,35,37,38,41,44,45,47,50,52,53,55,59,62,65,67,69,71]	
Experiences and needs regarding communication	“Even when the postoperative course was complicated, patients retained confidence in their healthcare providers and the healthcare system, as long as lines of communication remained open” [47].
	“Written or spoken communication should take place any time patient information has to be moved from one level of care to another or from a care setting to the program.” [26]
	“Healthcare professionals also discussed referral communication. Doctors explained that there were no structured processes to follow for information exchange during referrals: Yeah, there is no proper way of doing it… inpatients sometimes we must [refer] but as I told you we never had a structured format” [44].
Barriers to effective communication	“Patients reported they often considered the referral documentation to be of an administrative nature. Consequently, they did not always treat it with sufficient attention, resulting in documentation getting lost or transmittal being delayed” [37].
	“Primary care professionals described uncertainty in how to contact the hospitalist and having to speak with multiple persons before reaching the correct hospitalist. Hospitalists also described frustration about not having access to direct phone lines for primary care professionals” [35].

**Table 3.** Supporting data: quotes related to theme 3 and 4, and subthemes.

Theme 3: Patient and family involvement [13,21,23–25,31,36,37,39,41,45,47,52–56,58–60,63,69–71]	
Patient involvement	*“*Some of the participants believed that their family was involved in the process and had communicated with the healthcare staff. This was experienced differently; for some participants, not having to deal with planning the discharge seemed to be a relief, whereas others perceived it negatively and had been left feeling dissatisfied” [71].
Family involvement	“An interesting phenomenon is the transformation of family caregivers from supportive supporters to surrogates and advocates for care decisions, and the closeness of the relationship between health care workers and family caregivers even more than patients, which are mutually understood and commonplace” [32].
	“All carers explained that sustaining family relationships with the older adult required them to transition from being a family member to being a family carer” [31].
Theme 4: Individualized support and information provision [13,21,23–25,27–29,31,32,34,36,38–40,44–46,48–50,52,53,55–57,60–67,71,72]	
Experiences with individualized support	“Adjusting to life post-discharge involved learning to rely on others, a state of dependency that sometimes undermined their identity as an independent and productive individual. As one participant described, “It’s frustrating to watch my wife do everything. I have two kids and I feel like my wife [has] become a single mom with a third kid now because of having to do everything”’ [51].
	“Patient and carer participants described caring relationships with healthcare practitioners in terms of feeling cared for as a person, feeling included and respected” [31].
	“Frequently, patients also felt abandoned by the health care system once they left the hospital: “Because once they let you out the hospital, that’s it, you gone. You are no longer our responsibility” [65].
Facilitators and needs for individualized support	“Finally, patients reported feeling guilty and uncomfortable because although they needed their caregivers to assist them, they did not want to become a burden” [47].
	“Patients often described a ‘wait and see’ approach that, in some instances, delayed further investigation or precipitated a crisis: No, don’t ring the ambulance. I’ll be right till the morning. It’ll only be a bit of bruising” [36].
	“When they first came home, many had felt insecure about no longer being in the safe environment of the hospital” [65].
Barriers to individualized information exchange	“Discharge letters contained mistakes, leading to inadequate care provision after discharge. Incomplete or insufficient information delivered to family members on patients’ (in)abilities caused a feeling of being ill-prepared for the care tasks expected of them after hospital discharge” [66].
	“Many participants felt that the information was delivered in an inappropriate format: ‘It would have been nice to have somebody sit down with me and say this is what’s happened, this is why it’s happened, this is what you can expect” [69].

### Theme 1: Care coordination and continuity

Within this theme, two subthemes were identified: experiences and needs for care coordination and continuity and barriers and facilitators for care coordination and continuity.

### Experiences and needs for care coordination and continuity

Forty-four studies (9 high, 18 moderate, and 17 fair-quality studies) reported patients’ and professionals’ *experiences and needs for care coordination and continuity* [13,21–63].

Patients mainly shared their experiences and needs for continuity of aftercare, expressing a strong need for continuity of care after discharge from the hospital [23–25,31,40,45,46,51,53,56–60]. They highly valued any follow-up care provided after discharge and receiving a phone call from a healthcare professional and/or a follow-up visit during which they could ask questions. Nissim et al. illustrate this: “When feeling lost in the maze of their complex care, participants were extremely grateful when members of the medical team responded immediately by telephone or e-mail to questions about symptoms that developed at home or to problems in navigating services” [51]. Patients did not expect the healthcare professional who called them to be medically involved in their care. It was unclear to patients and healthcare professionals, however, whose responsibility it should be to call up the patient [45,46,51,56–58].

Patients’ experiences with hospital discharge varied widely. In some studies, patients described they were well prepared for discharge [23,31,45,52,53]. Patients of other studies experienced poor care coordination and that they had to arrange many things themselves, such as arranging walking aids or making appointments with primary care providers [21,23,31,32,41,45–51]. Patients expressed a strong desire to have someone to turn to (such as a case manager) for guidance during and after the hospital-to-home transition [23,27,31,32,34,36].

According to healthcare professionals, clear rehabilitation goals [23,26,31,33,36,38,39,61,64], knowing who coordinates care [23,30–36,39], and developing good relationships [23,29–31,60] with patients and colleagues were needed for sufficient care coordination.

### Barriers and facilitators for care coordination and continuity

Thirty-eight studies (7 high, 12 moderate, and 19 fair-quality studies) reported on organizational and systemic barriers and facilitators for care coordination [21–23,25–28,30,31,33–41,43–46,48,52–57,59,62,63,65–70].

Healthcare professionals identified the following barriers for care coordination after discharge. A lack of time [22,23,26,27,31,38,39,44], insurance issues, such as patients who cannot afford aftercare [22,35,38,41,43,48,54,70], the administrative burden [22,38] and not knowing who was responsible for what [22,34,35] were identified as significant barriers to guarantee care coordination. To illustrate this: “The nurses all felt a substantial responsibility for the patients, while they were in their care. However, they were unsure who had the overall responsibility for the transition process” [33].

Healthcare professionals also mentioned facilitators for care coordination after discharge. Early planning and organization of hospital discharge was one of the main facilitators for sufficient care coordination [21,23,26,30,31,36,40,44,45,53,55–57,59,65,67,68]. When healthcare professionals were sufficiently prepared for a patient’s discharge, for example by knowing the date of discharge in time, they were also better able to prepare their patients for it. Another facilitator for care coordination was the level of expertise of healthcare professionals – it was one of the cornerstones of providing high-quality care coordination [22,41,44,54,66,68].

Patients were more willing to continue their treatment with primary care professionals if they were familiar with them and had positive experiences with them [25,46,52,59].

### Theme 2: Communication

Two subthemes were identified in this theme: *experiences and needs regarding communication* and *barriers to effective communication.*

### Experiences and needs regarding communication

A total of 23 studies (4 high, 10 moderate, and 9 fair-quality studies) reported on experiences and needs regarding professional communication [13,22,23,25,29–32,34–44,47,52,54,62]. According to healthcare professionals, interdisciplinary information exchange affects professional communication [22,23,29,31,36–40,44,52,62]. There are various ways of exchanging information, such as discharge letters, phone calls or (secured) e-mails, which were sometimes inadequate. For example, discharge letters did not always reach the person to whom they were addressed, or healthcare professionals were not easily reached by phone. The following quote illustrates this: “General practice and primary care professionals often reported receiving late, inaccurate and/or unclear discharge letters and referrals” [29]. Hence, there is a need for more effective information transfer methods using standardized protocols or even a shared electronic patient record system for hospital and primary care [23,31,32,39–44,47].

### Barriers to effective communication

Eighteen studies (3 high, 8 moderate, and 7 fair-quality studies) reported barriers to effective communication [22,23,26,29,31,33,35,37,38,41,45,50,53,55,59,62,67,71]. In some studies, patients and healthcare professionals did not experience sufficient communication [23,26,31,37,45,59,67]. Examples of this include healthcare professionals not receiving information from colleagues, and patients not knowing who to contact in case of questions. To illustrate this: “Professionals reported they communicated mostly via discharge letters, resulting in a lack of personal contact, and pointed out the potential to miss out on crucial information” [37].

For healthcare professionals, lack of consistency in work schedules, data privacy issues such as not being able to share patient data with other healthcare professionals safely, and a mismatch in perceptions between healthcare professionals were barriers to effective communication [22,29,35,38]. A mismatch in perceptions was defined as different healthcare professionals perceiving a situation differently or having different thoughts and interests surrounding the situation. Some healthcare professionals mentioned that this may lead to misunderstandings and distrust between hospital and primary care professionals. This in turn creates parallel working cultures and might escalate into a “them versus us” approach, which is in turn detrimental for effective communication [22].

### Theme 3: Patient and family involvement

Twenty-four studies (5 high, 9 moderate, and 10 fair-quality studies) reported on patient and family involvement [13,21,23–25,31,36,37,39,41,45,47,52–56,58–60,63,69–71]. Patients and healthcare professionals reported their experiences and needs concerning patient and family involvement.

Patients felt it was important to be involved in making decisions regarding their transitional care plan [23,25,31,37,54,57,60,71]. Most patients had preferences regarding their own role. Assuming this role, however, was challenging as patients struggled to assert themselves [23,31]. Not being invited to participate in the decision-making regarding their transitional care plans was not always experienced negatively by patients. One patient indicated, for example: “I didn’t take part in the discussion about my care needs. I don’t remember that I was asked directly about what I wanted, but they didn’t do anything against my will, that’s for certain” [71].

Family support was deemed essential during the transition from hospital to home [21,23–25,31,36,37,39,45,53–55,58–60,71]. Caring for loved ones, however, affected the relationship between patients and their family members and could sometimes be stressful and challenging [23,24,30–32,47,56,60,69,70]. That is, balancing caregiving with daily tasks and concerns for a loved one contributes to heightened stress. To manage this, strategies like educating family members, particularly those with low literacy or older individuals [23,31], and providing timely information can ease their involvement.

### Theme 4: Individualized support and information provision

In this theme, three subthemes were identified: *experiences with individualized support, facilitators and needs for individualized support* and *barriers to individualized information exchange.*

### Experiences with individualized support

Thirty-one studies (7 high, 16 moderate, and 8 fair-quality studies) reported on experiences with individualized support [13,21,23–25,27–29,31,32,34,36,38,44–46,48–50,52,53,56,57,60,61,63,65,67,71,72]. Several studies emphasized the vulnerability of patients during the hospital-to-home transition. Patients sometimes felt powerless, weak, and overwhelmed by the number of healthcare professionals’ visits during their hospital stays [21,32,49,50,52,65]. When they arrived home, it was sometimes hard to adapt to daily life, to get back to work, or to resume other activities [49,51,56,72]. To illustrate this, a participant described: “The biggest challenge was fighting it so I would reach remission. But now, the biggest challenge is adapting to the fact that I’ve had it” [51]. Patients greatly appreciated feeling heard and indicated to prefer personalized over protocol-based care. Patients were satisfied if they received the support they needed, meaning that they received the right care at the right time, both physically and mentally. Patients felt that primary care providers were essential to achieving their goals at home [23,29,31,36,45,46,48,50,52,60].

In contrast to these positive experiences, patients also expressed negative experiences. Sometimes, patients feel they are a burden to their families and consuming valuable time family members may not always have [23–25,31,47,58,65,67,69]. To illustrate this, a participant said: “I wouldn’t tell [my daughter] if I deteriorated because she’s got a few medical problems as well…So I don’t want to burden her” [58]. Patients also feel they are a burden to healthcare professionals, leading to misunderstandings between patients and healthcare professionals. To illustrate, patients did not want to ask too much time from healthcare professionals. This, in turn, can lead to the feeling of being rushed out of the hospital [23,27,31,36,65]. Consequently, patients sometimes felt abandoned and lacking support [23,29,31,36,46,50,53,55,65]. When patients had questions after arriving home, for example, the hospital was not always reachable by phone for questions, which was perceived as very frustrating. Some patients even called an ambulance to get answers to their questions [23,31,34,36,38,51,53,57,67]. Sometimes, patients declined the offered assistance [36,57,71], such as recommendations to see a dietitian, as they preferred managing nutrition at home. Patients’ desire for various types or amounts of support underscores the necessity for personalized support plans for patients and their families.

### Facilitators and needs for individualized support

A total of 24 studies (2 high, 12 moderate, and 10 fair-quality studies) reported on facilitators and needs for individualized support [23,24,31,32,36,40,44–48,50,52,53,55,58–60,62,63,65,67–69]. Patients strongly emphasized the importance of individuality and independence. While in the hospital, they were not always independent in their activities, making it crucial for them to regain independence during the transition from hospital to home [23,31,58,60,68]. To patients, feeling included and respected as well as the possibility of thanking healthcare professionals after discharge from the hospital were seen as facilitators for individualized support [23,31,44,46].

A safe environment for patients is a facilitator for personalized support. For example, some patients believed the hospital was the best place to recover. Other patients expressed unfamiliarity with the hospital environment and, therefore, staying in the hospital was confusing, as they missed their daily routine [40,46,65].

### Barriers to individualized information provision

A total of 25 studies (3 high, 13 moderate, and 9 fair-quality studies) reported on barriers to effective information provision especially related to the quantity, clarity, and correctness of the information provided to patients [21,23,31,32,36,37,41,44–51,53,55,57–59,65,67,69,71,73]. Several studies reported that patients received information that was unclear to them. Information was also often incorrect, unavailable, or provided at the wrong moment [21,23,31,37,41,44,46,48–51,53,57,59,64,65,67,69,71,73]. Patients also indicated they typically received a lot of information at once, which overwhelmed them [15,23,31,32,41,44,45,51,53,57–59,64,67]. The following quote illustrates that information provided during the transition process does not always stick: “You read it but you don’t really take it in…like there were things that were said by the doctors that my wife heard and remembered, and I didn’t get” [51]. Studies mentioned a strong need for appropriate, honest, and detailed information that is understandable to patients and their families. This information should be provided in a timely manner, and there should be an opportunity to ask questions [36,37,41,44–47,55,73].

Not daring to ask questions is also a barrier in information provision. Patients might often think, for example, that their problems are not severe enough to seek help [36,46,65]. Moreover, they often do not dare to ask questions, because they think healthcare providers are too busy to answer them. To illustrate, a participant said: “You can’t ask doctors or nurses questions even if you don’t understand because they don’t keep still long enough” [65]. Trust also plays a significant role in this subtheme. That is, patients only dare to ask questions if they trust healthcare professionals [46,52,55,59,67].

The secondary analysis showed that there was a well-balanced distribution of the four themes across the countries involved in the studies included (see Supplementary File 2). This secondary analysis also revealed that non-Western countries were underrepresented in our study.

## Discussion

This systematic meta-synthesis is the first to provide a comprehensive overview of experiences with hospital-to-home transitions from the perspective of patients, family members, and (allied) healthcare professionals. We adopted a novel approach by combining the experiences of various stakeholders to obtain more comprehensive information on hospital-to-home transitions from a diverse range of perspectives. This information can inform healthcare professionals, researchers, and policymakers who wish to develop interventions to optimize care coordination and continuity after hospital discharge, thus enhancing health-related outcomes and reducing costs. We identified four themes that transcend the results of the current literature. This study adds information on how patients, family members, and healthcare professionals experience hospital-to-home transitions and what they need to optimize the experience of this transition. These themes *were: care coordination and continuity, communication, patient and family involvement, and individualized support and information exchange, and* were supported by high-quality evidence.

The first theme – care coordination and continuity – illustrates the importance of supporting patients in arranging their follow-up care to allow them to pursue their recovery with primary care professionals in a timely manner. Our findings suggest that patients would benefit from a phone call or follow-up appointment from the hospital. Patients find it easier to receive follow-up care from primary care professionals with whom they are already familiar. The feasibility and effectiveness of post-hospital discharge telephone follow-up have previously been assessed in a study by Harrison et al. [74]. They found telephone follow-up to be feasible in reaching older adults and effective in identifying post-hospital problems experienced by older adults. However, further research should focus on the feasibility and effectiveness of follow-up interventions in other populations, particularly those with allied healthcare needs, as they require prompt follow-up care after discharge. In our study, healthcare professionals also expressed a need for more timely preparation of a patient’s discharge, more explicit multidisciplinary rehabilitation goals, approachable and available colleagues, and precisely knowing each other’s roles and who is responsible for coordinating follow-up care. In line with our findings, previous research on user experiences during the transition from hospital to home in older individuals identified that clarifying ‘who is taking care of what’ is crucial for effective collaboration among healthcare providers [75,76]. Other studies have previously demonstrated the benefits of transmural collaboration within an interdisciplinary network of (allied) healthcare professionals [66,77].

The second theme – communication – illustrates experiences, needs, and barriers regarding communication. An interesting finding was the occurrence and consequences of mismatched perceptions about referral information. Hospital and primary care healthcare professionals have different thoughts and expectations regarding the content of information they exchange, which may negatively affect communication between them and, in turn, affect the quality of care provided. Researchers have previously suggested using shared electronic patient records for hospitals and primary care settings to enhance interprofessional communication [35,78]. Jones et al. found that both hospital and primary care professionals proposed shared electronic medical records as a solution to enhance information exchange in care coordination [35]. This finding was corroborated by Munchof et al. However, each care setting generally uses a different electronic system, which poses an implementation challenge [79]. Together with our results, this implies a need to develop innovative and more effective solutions to close the communication loop between hospital and primary care professionals.

The third theme – patient and family involvement – illustrates the importance and challenges of involving patients and families in making decisions regarding hospital-to-home transitions. From our results and other sources, it is clear that patients and their families experience shifting relationships when families have to care for their loved ones [13,80]. Therefore, involving patients and families at all stages of the hospital-to-home transition is essential. Naylor et al. outlines strategies for implementing patient and family involvement in hospital-to-home transitions, such as comprehensive goals assessment, progress monitoring, demonstrating respect, and evaluating engagement levels [80]. Additional research is needed to explore the practical implications of implementation of such strategies. We recommend involving patients and informal caregivers in designing, implementing and evaluating these strategies to ensure sustainable change and improvement in transitional care.

The fourth theme – Individualized support and information provision - illustrates experiences, needs, facilitators and barriers regarding personalized strategies of providing support and information to patients. Our results stress that patients can be vulnerable at the moment of hospital discharge and may need personalized support. Another result was the importance of how information is provided to patients, which was often unclear, incorrect, or overwhelming for patients. Menichetti et al. outlines cognitive strategies, including simplification, clustering, ordering, repetition, and teach-back, as beneficial tools for healthcare professionals to optimize information provision to patients transitioning from hospital to home. To effectively employ these strategies, further effort should focus on educating healthcare professionals on these topics [81].

### Strengths and limitations

This meta-synthesis provides extensive insight into current experiences with hospital-to-home transitions and could offer important leads for developing and implementing future transmural care pathways for patients needing complex care interventions after hospitalization. We included a large number of studies conducted in fourteen different countries, increasing our findings’ transferability and the richness of our results. Our secondary analysis shows that our results may apply to different healthcare systems in different parts of the world. It should be noted that some non-Western countries, such as those in the southern hemisphere, are underrepresented in the results of our study.

This meta-synthesis was conducted following a rigorous methodology. An extensive search was performed, followed by a thorough critical selection and appraisal method. Moreover, a snowball search was conducted to include as much available data as possible. Thereby, we excluded insufficient-quality articles according to the rules of Butler et al. with a CASP score <6.0, and all themes we identified were supported by high-quality studies with a CASP score >9.0, implying a high level of evidence for each theme [16]. Many articles were identified, and we found indications of inductive thematic saturation, which appeared during the analysis process when no new themes were emerging [82].

However, some limitations to our study can be identified. First, due to the large number of researchers participating in the selection process, uniformity in article selection was possibly affected negatively. To minimize this selection bias, we used sample checks and held meetings during the screening phase to monitor and guide the process. Second, the perspective of family caregivers is minimally represented in our meta-synthesis. This may be because the opinions of patients and family members are often presented in a combined manner. Consequently, our results mainly represent the perspectives of patients and healthcare professionals, which provided us with a broad view from different perspectives. Third, we recognize that there may be overlap in the information presented in different subthemes due to the complexity of the data. However, reporting experiences, needs, barriers, and facilitators distinctly provided a more nuanced and comprehensive understanding of the studied phenomena.

## Conclusion

This study revealed both positive and negative experiences of patients, family members, and professionals, as well as barriers and facilitators regarding hospital-to-home transitions across various care settings, countries, and populations. Tailored information and support are prerequisites for a seamless transition and an optimal recovery after hospital discharge. Particular attention should be paid to the amount, the mode of communication and the timing of information and support provision to patients and their families. On the other hand, healthcare professionals should communicate more effectively within and across care settings to guarantee optimal coordination. Individual professional roles should be clarified to ensure continuous collaboration and, thus, continued high-quality care provision after hospital discharge. When ensuring continuity of care through the design of integrated transitional care interventions, these findings should be considered, and strategies implemented. Novel research methods, such as implementation research, can be used to investigate how best to implement measures addressing the barriers and facilitators identified in this study.

## Supplementary Material

Supplemental Material

## Data Availability

Data will be available upon request via DataVerseNL https://doi.org/10.34894/YYIIY6.

## Appendix

**Table A1.** Searches.

Database(s): Ovid MEDLINE(R) ALL 1946 to June 19, 2024		
Search Strategy:		
#	Searches	Results
1	exp long term care/ or exp comorbidity/ or exp multimorbidity/	158,965
2	((complex or "long term" or chronic or multidisciplinary) adj3 (care or treat* or therapy)).ti,ab,kf.	301,516
3	((complex or "high risk") adj3 (patient* or medically or adult*)).ti,ab,kf.	132,506
4	(multiple adj3 (chronic adj3 (condition* or disease* or disorder* or ill* or patholog* or "health problem*"))).ti,ab,kf.	4083
5	(comorbidit* or "co morbidit*" or multimorbidit* or (("intercurrent" or concurrent) adj2 (illness* or disorder* or disease* or condition* or patholog* or "health problem*"))).ti,ab,kf.	249,158
6	(polymorbidit* or Plurimorbidit* or ((multiple or polypathic) adj2 (condition* or disease* or disorder* or illness* or patholog* or "health problem*"))).ti,ab,kf.	43,934
7	or/1-6	789,951
8	transitional care/ or exp "Continuity of Patient Care"/ or exp Patient Care Planning/ or exp Case Management/	363,262
9	((transition* or discharge or postdischarge or handover or "follow up" or stepdown or "step down" or multidisciplinary) adj3 (care or treat* or therapy or coordinat*)).ti,ab,kf.	108,209
10	((plan* or admission or discharge or postdischarge) adj3 (coordinat* or contin* or manag* or process*)).ti,ab,kf.	72,495
11	((handover or handoff* or "hand over" or "hand off*" or signout* or "sign out*" or signover or "sign over") adj3 (patient* or plan* or coordinat* or manag* or program*)).ti,ab,kf.	1312
12	(care adj3 (coordinat* or plan* or manag* or goal* or contin* or process*)).ti,ab,kf.	152,397
13	(case adj3 (manag* or plan* or coordinat* or program*)).ti,ab,kf.	33,918
14	or/8-13	663,960
15	exp implementation science/ or exp Systems Analysis/ or exp Program Evaluation/ or exp Evaluation Study/ or exp Health Knowledge, Attitudes, Practice/	566,154
16	((system* or program* or process*) adj3 (analy* or integrat* or evaluat* or implement*)).ti,ab,kf.	423,384
17	(implement* or evaluat* or coordinat* or strateg* or barrier* or block* or obstacle* or hinder* or constrain* or facilitat* or incentiv* or challenge* or enabler*).ti,ab,kf.	9,070,179
18	(Health adj3 (Knowledge or attitude* or practice*)).ti,ab,kf.	58,830
19	or/15-18	9,492,755
20	exp Patient Readmission/ or exp Mortality/ or exp "Quality of Life"/ or exp "Costs and Cost Analysis"/ or exp Self Efficacy/ or exp Outcome Assessment, Health Care/ or exp "Quality of Health Care"/ or exp Quality Indicators, Health Care/ or exp "Surveys and Questionnaires"/ or *Hospitalization/	8,764,626
21	(rehospitalization or hospitali* or ((patient* or hospital* or "30 day" or "thirty day" or unplanned) adj3 (readmission* or re admission))).ti,ab,kf.	387,264
22	(mortal* or ((death or fatality) adj2 (rate* or frequenc*))).ti,ab,kf.	1,089,674
23	(Self adj2 (Efficacy or concept)).ti,ab,kf.	52,195
24	(Outcome adj3 (assess* or patient* or clinical* or "health Care")).ti,ab,kf.	284,467
25	(Survey* or questionnaire* or cost*).ti,ab,kf.	2,225,187
26	(QOL or hrql or hrqol or (Quality adj2 (Healthcare or care or life))).ti,ab,kf.	508,587
27	or/20-25	10,328,172
28	exp Adult/ or exp Aged/ or exp Geriatrics/	8,120,550
29	(adult* or senior* or aged or elder* or geriatri* or ((old* or mature*) adj2 (people* or subject* or patient* or age* or men or male* or wom?n or female* or population* or cohort* or person*))).ti,ab,kf.	3,168,888
30	or/28–29	9,523,688
31	7 and 14 and 19 and 27 and 30	17,747
32	review.pt.	3,338,721
33	case reports.pt.	2,411,891
34	((exp animals/ or exp veterinary medicine/ or animal*.jw.) not exp humans/) or (experiment* model* or animal* or monkey* or sheep or ?ovine or lamb* or goat* or pig* or swine or porcine or pup* or dog* or canine or bitch* or beagle* or feline or rodent* or rabbit* or rat or rats or mouse or murine or mice).ti,kf.	5,845,932
35	((Palliative or "end of life") adj3 care).ti,kf.	37,182
36	or/32–35	11,209,875
37	31 not 36	15,368
Database(s): Embase 1974 to 2024 June 19		
Search Strategy:		
#	Searches	Results
1	exp long term care/ or exp comorbidity/ or exp multiple chronic conditions/	2,808,078
2	((complex or "long term" or chronic or multidisciplinary) adj3 (care or treat* or therapy)).ti,ab,kf.	424,465
3	((complex or "high risk") adj3 (patient* or medically or adult*)).ti,ab,kf.	223,352
4	(multiple adj3 (chronic adj3 (condition* or disease* or disorder* or ill* or patholog* or "health problem*"))).ti,ab,kf.	5290
5	(comorbidit* or "co morbidit*" or multimorbidit* or (("intercurrent" or concurrent) adj2 (illness* or disorder* or disease* or condition* or patholog* or "health problem*"))).ti,ab,kf.	449,925
6	(polymorbidit* or Plurimorbidit* or ((multiple or polypathic) adj2 (condition* or disease* or disorder* or illness* or patholog* or "health problem*"))).ti,ab,kf.	62,366
7	or/1-6	3,462,976
8	exp clinical handover/ or exp patient care planning/ or exp case management/	54,286
9	((transition* or discharge or postdischarge or handover or "follow up" or stepdown or "step down" or multidisciplinary) adj3 (care or treat* or therapy or coordinat*)).ti,ab,kf.	175,311
10	((plan* or admission or discharge or postdischarge) adj3 (coordinat* or contin* or manag* or process*)).ti,ab,kf.	94,468
11	((handover or handoff* or "hand over" or "hand off*" or signout* or "sign out*" or signover or "sign over") adj3 (patient* or plan* or coordinat* or manag* or program*)).ti,ab,kf.	2192
12	(care adj3 (coordinat* or plan* or manag* or goal* or contin* or process*)).ti,ab,kf.	215,411
13	(case adj3 (manag* or plan* or coordinat* or program*)).ti,ab,kf.	50,005
Database(s): Embase 1974 to 2024 June 19		
Search Strategy:		
#	Searches	Results
14	or/8-13	531,816
15	exp implementation science/ or exp system analysis/ or exp program evaluation/ or exp evaluation study/	133,241
16	((system* or program* or process*) adj3 (analy* or integrat* or evaluat* or implement*)).ti,ab,kf.	540,126
17	(implement* or evaluat* or coordinat* or strateg* or barrier* or block* or obstacle* or hinder* or constrain* or facilitat* or incentiv* or challenge* or enabler*).ti,ab,kf.	11,786,761
18	(Health adj3 (Knowledge or attitude* or practice*)).ti,ab,kf.	70,103
19	or/15-18	12,013,423
20	exp hospital readmission/ or exp mortality/ or exp "quality of life"/ or exp "cost"/ or exp self concept/ or exp evaluation study/ or exp outcome assessment/ or exp health care quality/ or exp questionnaire/ or *hospitalization/	7,037,525
21	(rehospitalization or hospitali* or ((patient* or hospital* or "30 day" or "thirty day" or unplanned) adj3 (readmission* or re admission))).ti,ab,kf.	636,455
22	(mortal* or ((death or fatality) adj2 (rate* or frequenc*))).ti,ab,kf.	1,608,270
23	(Self adj2 (Efficacy or concept)).ti,ab,kf.	60,437
24	(Outcome adj3 (assess* or patient* or clinical* or "health Care")).ti,ab,kf.	461,295
25	(Survey* or questionnaire* or cost*).ti,ab,kf.	2,962,428
26	(QOL or hrql or hrqol or (Quality adj2 (Healthcare or care or life))).ti,ab,kf.	784,602
27	or/20-26	9,364,753
28	exp adult/ or exp adult/ or exp geriatrics/	11336,,335
29	(adult* or senior* or aged or elder* or geriatri* or ((old* or mature*) adj2 (people* or subject* or patient* or age* or men or male* or wom?n or female* or population* or cohort* or person*))).ti,ab,kf.	4,367,803
30	or/28-29	12,796,657
31	7 and 14 and 19 and 27 and 30	45,091
32	exp "review"/	3,284,134
33	exp case report/	3,011,222
34	((exp animals/ or exp veterinary medicine/ or animal*.jw.) not exp humans/) or (experiment* model* or animal* or monkey* or sheep or ?ovine or lamb* or goat* or pig* or swine or porcine or pup* or dog* or canine or bitch* or beagle* or feline or rodent* or rabbit* or rat or rats or mouse or murine or mice).ti,kf.	5,951,680
35	((Palliative or "end of life") adj3 care).ti,kf.	54,539
36	exp conference abstract/	2,317,960
37	or/32-36	13,777,808
38	31 not 37	26,335
