# Supplementary material for: Experiences with hospital-to-home transitions: perspectives from patients, family members and healthcare professionals. A systematic review and meta-synthesis of qualitative studies
Source: Disabil Rehabil. 2024 Aug 5;47(7):1644–58. doi: 10.1080/09638288.2024.2384624 (PMC11974919; doi:10.1080/09638288.2024.2384624)
Supplement: Supplemental Material [file IDRE_A_2384624_SM8107.zip › Supplementary file 1 study and participant characteristics.docx]

| Table: Study and participant characteristics | | | | | | |
| --- | --- | --- | --- | --- | --- | --- |
| **Author, year, country** | **Aim** | **Patient population** | **Occupation professionals** | **Work setting professionals** | **Demographics N [Mean age(range), sex M/F]** | **Design and data collection method** |
| Allen et al., 2020, Australia | To evaluate healthcare practitioners’ perceptions of the feasibility and acceptability of a communication tool to communicate with patients during transition from acute care to a community setting | Older patients with chronic conditions | Nurse practitioner  Physiotherapist Social worker  Occupational therapist  Pharmacist | Primary care and hospital | Healthcare professionals: 22[NA (20-70+), NA] | Exploratory descriptive qualitative design  Semi-structured interviews |
| Allen et al., 2018, Australia | How do older people and their carers/families as care recipient service users experience discharge and transitional care across the trajectories of acute, subacute and community care? | Older patients and their carers | NA | NA | Patients: 20 [78.9 (45-94)4/16] | Exploratory descriptive qualitative design  Semi-structured interviews |
| Agerholm et al., 2023, Sweden | To examine healthcare professionals’ perceptions on barriers and facilitators for care coordination for older patients with complex health and social care needs being discharged from hospital | Older patients with complex health and social care needs | Nurses | Primary care and hospital | NA | Comparative study  Semi-structured interviews |
| Allen et al., 2022, Australia | To co-design a communication tool to guide conversations about transitional care needs between healthcare practitioners and patients returning to the community | Older patients with chronic conditions | Nurse practitioner  Social worker  Medical practitioner  Physiotherapist  Occupational therapist  Pharmacist  Case manager | Primary care and hospital | Patients: 19[86.2(72-94), 8/11]  Carers: 7[68.9(45-88), 2/5]  Healthcare professionals: 48[44(23-64), 7/40] | Action research  Semi structured interviews and focus groups |
| Allen et al., 2022, Australia | To understand enablers and barriers to the inclusion of carers in transitional care from carers' and healthcare practitioners' perspectives | Older patients with chronic conditions | Nurse practitioner  Physician  Social worker  Pharmacist  Occupational therapist  Educator | Inpatient rehabilitation center | Carers: 17[NA (44-80), 3/14]  Healthcare professionals: 8[NA (30-59), 1/7] | Exploratory descriptive qualitative design  Semi structured interviews and focus groups |
| Antony, et al., 2018, USA | To explore inpatients’ perceptions about factors contributing to their rehospitalization and their recommendations to reduce this risk | Older veterans with a low socioeconomic status | NA | NA | Patients: 18[71.6(57-90), 17/1] | Thematic qualitative study  Interviews |
| Arora et al., 2010, USA | To report patients’ experiences with problems after hospital discharge and investigate whether primary care professionals were aware of their hospitalization | Older patients | NA | NA | Patients: 64[73(NA), 20/44] | Prospective mixed methods study  Telephone interviews |
| Backman et al., 2018, Canada | To engage older adults and their family members in the detailed exploration of their experiences during transitions across healthcare settings and identify potential areas for future interventions | Older patients with multiple chronic conditions | NA | NA | Patients: 9[77.6(56-94), 3/6]  Carers: 8[NA(NA), 1/7] | Qualitative study using participatory visual narrative methods  Narrative photo walks |
| Baxter et al., 2020, UK | To explore staff perceptions of how high performing general practice and hospital specialty teams deliver safe transitional care to people as they transition from hospital to home | Older patients | Physician  Nurse practitioner  Healthcare assistant  Receptionist  Administrator  Allied health professional  Discharge coordinator | Primary care, community care, and hospital | Healthcare professionals: 157 [NA(NA), NA] | Qualitative design, not further specified  Semi-structured focus group |
| Brez et al., 2009, Canada | To explore primary care physician perspectives and concerns related to reassuming responsibility for diabetes care after referral to a specialized diabetes center | Patients with diabetes | Physician | Primary care | Healthcare professionals: 22[NA(NA), NA] | Qualitative design, not further specified  Focus groups |
| Brooke et al., 2018, USA | To gain a better understanding of the factors that influence how patients and caregivers  perceive care coordination during transitions of surgical care | Surgical patients | NA | NA | Patients: 90[62.5(NA), 52/38]    Carers: 24[63.2(NA), 10/14] | Qualitative design, not further specified  Focus groups |
| Crawshaw et al., 2021, UK | To investigate the perceptions and early experiences of patients transitioning from hospitals in the UK and USA to home | Patients with acute coronary syndrome | NA | NA | Patients: 17[62.6(43-81), 13/4] | Exploratory qualitative study  Semi-structured telephone interviews |
| Cobley et al., 2013, UK | To investigate patients' and carers' experiences of Early Supported Discharge services and inform future Early Supported Discharge service development and provision | Stroke patients and carers | NA | NA | Patients 27 [69.85 ± 13.42 years]    Carers 15 [72.79 ± 14.10] | Thematic analysis  Semi-structured interviews |
| Davis et al., 2012, USA | To understand care transitions from the perspective of diverse healthcare professionals and identify recommendations for process improvement | NA | Clinicians, care teams and administrative staff | Inpatient general medicine services in an urban and academic hospital, outpatient clinics and Medicaid. | 15 [43 years] | Cross-sectional qualitative study  Focus groups |
| Doos, et al., 2015, UK | To explore the experiences of multimorbid patients and their carers on hospital discharge | Patients with heart failure and COPD | NA | NA | Patients: 6 [79(62-91), 3/3]    Carers: 5 [NA(NA), 1/4] | Mixed-methods study  Interviews |
| Dutton et al., 2014, Canada | To explore patients’ expectations and experiences surrounding discharge from a specialized diabetes center back to primary care | Patients with diabetes | NA | NA | Patients: 12[NA(NA), 5/7] | Qualitative design, not further specified  Semi structured interviews |
| Fox et al., 2023, UK | To identify organizational processes that can be targeted to reduce variation in service provision and improve patient care | Patient who underwent surgery for hip fracture | Physician  Nurse practitioner  Physiotherapist Occupational therapist  Manager  Theater practitioner  Coordinator | Hospital | Healthcare professionals: 40[NA(NA), NA] | Qualitative design, not further specified  Interviews |
| Groene et al., 2012, Spain | To explore handover practices at discharge and to focus on the patients’ role in handovers and on the potential additional risks for vulnerable patients | Patients with chronic conditions | Physician  Nurse practitioner | Primary care and hospital | Patients: 12[NA (63-100), 5/7)  Health professionals: 22[NA(27-59), 8/14] | Qualitative design, not further specified  Interviews |
| Grootel et al., 2024, The Netherlands | To gain insight into patients' experiences, perceptions, and needs regarding hospital-to-home transition, focusing on allied healthcare | Patients with complex care needs | NA | NA | Patients: 19 [50(NA)12/7] | Qualitative semi-structured interviews |
| Gustafsson et al., 2013, Australia | To investigate the experiences and expectations of  people with stroke, during their transition from hospital to home, after participating in a novel  inpatient outreach program | Stroke patients | NA | NA | 7 patients [61 NA] | Qualitative approach  Semi-structured interviews |
| Harvey et al., 2017, Australia | To describe the care transition experiences of people who transfer between subacute and primary care, and to identify factors that influence these experiences | Older patients | Nurse practitioner  Occupational therapist  Social worker  Physiotherapist  Physician  Coordinator | Primary care, community care, and hospital | Patients: 19[82.8(64-95), 12/7]    Healthcare professionals: 23[NA(NA), NA] | Exploratory, longitudinal case study  Semi structured interviews and focus groups |
| Humphries et al., 2019, India | To investigate patient and healthcare provider knowledge, attitudes and barriers to handover and healthcare communication during inpatient care | Patients with chronic conditions | Physician  Nurse practitioner  Pharmacist  Administrator | Hospital | Patients: 20[54.5(25-72), 10/10]    Healthcare professionals: 21[39.3(25-55), 15/6] | Qualitative design, not further specified  Semi structured interviews |
| Jepma et al., 2021, The Netherlands | To explore the experiences of participants who received a nurse-coordinated transitional care intervention | Older cardiac patients | NA | NA | Patients: 16[82.4(71-89), 8/8] | Generic qualitative approach  Semi-structured interviews |
| Jones et al., 2015, USA | To understand the challenges in coordination of care, as well as potential solutions, from the perspective of hospitalists and primary care physicians | Not specified | Physician  Physician assistant  Pharmacist | Primary and hospital | Healthcare professionals: 58[NA(NA), 29/29] | Exploratory qualitative study  Focus groups |
| Kable et al., 2015, Australia | To report acute community and residential care health professionals' perspectives on the discharge process and transitional care arrangements for patients and their carers | Patients with dementia | Physician  Nurse practitioner  Allied health professional  Administrator | Community care and hospital | Healthcare professionals: 33[NA(NA), NA] | Qualitative descriptive study design  Focus groups |
| Kangovi et al., 2014, USA | To explore low socioeconomic status patients’ perceptions of hospitalization, discharge and post-hospital transition in order to generate hypotheses and identify common experiences during this transition | Patients with a low socioeconomic status | NA | NA | Patients: 65[51.9(18-93), 24/41] | Exploratory qualitative design  Semi-structured interviews |
| Kelly et al., 2016, USA | To understand the expectations and perceptions of postoperative inpatients regarding transition from hospital to home in an effort to reduce patient burden | Patients who underwent surgery for colorectal cancer | NA | NA | Patients: 16[58(29-94), 8/8] | Qualitative design, not further specified  Interviews |
| Kimmel et al., 2016, Australia | To investigate orthopedic trauma patient experiences of discharge from the acute hospital and transition back into the community | Orthopedic trauma patients | NA | NA | 94 patients [18-64 NA] | Qualitative design  In depth interviews |
| King et al., 2022, Australia | To explore older people and their family members' perspectives on how discharge plans assisted self-management of their chronic conditions, their recognition of deterioration and when to seek treatment/re-attend hospital | Older patients with chronic conditions | NA | NA | Patients:19[NA(NA, NA]    Carers:8[NA(NA), NA] | Qualitative exploratory design  Interviews |
| Kokorelias et al., 2023, Canada | To explore the goals important to older adults, their caregivers, and care providers as they transition from hospital-to-home and how, if a patient navigation can enable goals of care | Older adults and their caregivers | Administrator  Manager Social worker Patient navigator Registered nurse Occupational therapist Recreational therapist Physiotherapist Physician Psychogeriatric therapist | Hospital | Patients: 9[79(68-95)6/10]  Family carers: 5[60(50-73)2/3]  Healthcare professionals: 48 | Qualitative semi-structured interviews |
| Kokorelias et al., 2023, Canada | To report on older adults’ and caregivers’ experiences of receiving services from a hospital-to-home patient navigation program. | Older adults and their family caregivers | NA | NA | Patients: 9 [79(68-95)6/10]  Family carers: 5[60(50-73)2/3] | Telephone interviews |
| Lilleheie et al., 2020, Norway | This study explores their experiences of the quality of the health ser-vices in hospital and the first 30 days at home after discharge | Older patients | NA | NA | 18 patients [NA(NA), NA] | Semi-structured interviews |
| Lou et al., 2016, Denmark | To investigate how mild stroke patients’ and their  partners’ experience and manage everyday life | Early supported discharge patients and partners | NA | NA | 22 patients [NA(NA), NA]  18 partners [NA(NA), NA] | Qualitative interviews |
| Major et al., 2021, The Netherlands | To investigate the feasibility of an interdisciplinary rehabilitation program designed for patients with Post Intensive Care Syndrome | Former ICU patients | Physiotherapist | Primary care | Healthcare professionals: 11[NA(NA), NA] | A mixed method, nonrandomized, prospective feasibility study  Focus group |
| Major et al., 2019, The Netherlands | To explore hospital discharge experience and to identify perceived barriers and enablers for a positive transition experience from hospital to home or rehabilitation facility as perceived by survivors of critical illness and their families | Survivors of critical illness and their relatives | NA | NA | 22 patients [53(11)]  13 relatives. | Grounded theory  Semi-structured interviews |
| Maximos et al., 2024, Canada | To examine the perspectives of support staff, health care professionals, and care coordinators working in or referring to a community-based, slow-stream rehabilitation, hospital-to-home transition program | Hospitalized patients | Support staff  Health care professionals  Care coordinators | Hospital and community care | 23 healthcare professionals [NA(NA), NA] | Interviews and focus groups |
| McFadden et al., 2022, USA | To better understand the challenges in the discharge transition of care | Surgical patients | Physician  Nurse practitioner | Hospital | Patients: 10[57(NA), 7/3]    Healthcare professionals: 10[NA(NA), NA] | Qualitative design, not further specified  Interviews |
| Nissim et al., 2014, Canada | To provide insight into the experience of patients in the transition from inpatient to ambulatory care | Patients with acute myeloid leukemia | NA | NA | Patients: 35[49(26-71), 22/13] | Qualitative design, not further specified  Interviews |
| Oravec et al., 2022, Canada | To investigate patients, caregivers, and healthcare providers' perceptions of the discharge process after cardiac surgery | Cardiac surgery patients | NA | NA | Patients: 16[69(52-85), 7/9]    Carers: 8[NA(NA), NA] | Mixed-methods study  Focus groups |
| Park et al., 2022, Korea | To investigate barriers and possible solutions for transfer planning of complex older patients in this study. | Older patients | Physician | Hospital | Healthcare professionals: 12[NA(NA), NA], 4/8] | Qualitative descriptive designed study  Focus groups |
| Persson et al., 2022, Denmark | To identify and analyze healthcare professionals’ perspectives and approaches to care coordination across sectors when older people are acutely hospitalized | Older patients | Physician  Nurse practitioner | Primary care and hospital | Healthcare professionals: 13[NA(NA), NA] | Qualitative design, not further specified  Semi structured interviews |
| Petersen et al. 2019, Denmark | To explore how the hospital and home care nurses talk about and experience cross-sectoral collaboration related to the transitional care of frail older patients | Medical and surgical patients | Registered nurses | Hospital, municipalities | 27 hospital nurses [NA(NA), NA]  52 home care nurses [NA(NA), NA] | Qualitative design  Focus groups  Observations |
| Prinjha et al., 2009, UK | To explores patients' perceptions and experiences of post ICU follow-up services | Former ICU patients | NA | NA | Patients: 34[52.1(23-76), 20/14] | Qualitative design, not further specified  Semi structured interviews |
| Rustad et al., 2016, Norway | To explore how patients ≥80 years of age experienced the care transition from hospital to municipal health care services | Older patients | NA | NA | 14 patients [88 (NA), NA] | Descriptive explorative design  Semi-structured interviews |
| Sandlund et al., 2024, Sweden | To explore home rehabilitation therapists’ experiences of supporting physical exercise after acute hospitalization, including exercise programs initiated during hospital stay | General patients | Physiotherapists  Occupational therapists  Managers | Rehabilitation therapy services | 12 healthcare professionals 47[(29–59),2/10] | Qualitative interviews |
| Shannon et al., 2022, UK | To assess intervention acceptability, identify implementation tools, and further develop the intervention | Older patients | NA | NA | 25 patients [84 (NA), NA] | Qualitative formative evaluation  Semi-structured interviews |
| Strunin et al., 2007, USA | To understand the phenomenon of frequent rehospitalization from the perspective of discharged patients | Patients with chronic conditions | NA | NA | Patients: 21[45.5(18-79), 10/11] | Qualitative design, not further specified  Semi structured interviews |
| Sun et al., 2023, China | To explore barriers and facilitators in the transition of care from hospital to home for older adults in China from the perspectives of older patients with chronic diseases and healthcare professionals. | Older adults with chronic conditions | General practitioners and nurses | Primary care  Hospital | Patients: 10 [74.3(NA), NA]  Caregivers: 9 [32.8(NA), NA] | Qualitative design, not further specified  Interviews |
| Swinkels et al., 2009, UK | To access the perspectives of older people on their experience of delayed transfer from acute hospital settings | Older patients who were acutely hospitalized | NA | NA | Patients: 23 [82.4(74-90), 11/12] | Qualitative design, not further specified  Semi structured interviews |
| Thys et al., 2024, Belgium | To explore the views of patients and healthcare providers on current rehabilitation after lumbar fusion surgery to fuel the development of a novel rehabilitation care pathway | Lumbar surgery patients | Physiotherapist  Surgeon  Nurse  General practitioner  Anaesthetist  Psychologist Occupational therapist | (non)academic settings | 5 patients [NA(NA), NA]  31 healthcare professionals [NA(NA), NA] | In depth interviews |
| Verweij et al., 2021, The Netherlands | To evaluate healthcare professionals' performance and treatment fidelity in the Cardiac Care Bridge nurse-coordinated transitional care intervention | Older cardiac patients | Nurse practitioner  Physiotherapist | Primary care, community care, and hospital | Healthcare professionals: 19[40.8(23-62), 2/17] | Mixed methods process evaluation  Semi-structured interviews |
| Vogel et al., 2024, Canada | To identify actionable themes to create a more efficient discharge process tailored specifically to the vascular surgery population | Surgical patients | Physicians  Nurses  Nurse practitioners  Social worker  Dietitian  Pharmacist | Tertiary center | Healthcare professionals: 33[NA(NA), NA]  Patients: 5[NA(NA), NA] | Focus groups |
| Witt et al., 2024, Canada | To explore health care providers’ experiences related to transitions in care from inpatient rehabilitation to the community for patients with limb loss | Patients with limb loss | Care Coordinator  Nurse  Clinician  Physician  Prosthetist  Rehabilitation therapist | Rehabilitation hospital | Healthcare professionals: 14[NA(NA), 4/10] | Semi-structured interviews |
| Abbreviations: N=number, M=male, F=female, NA=not applicable, COPD=chronic obstructive pulmonary disease, ICU=intensive care unit | | | | | | |
