# Supplementary material for: Experiences with hospital-to-home transitions: perspectives from patients, family members and healthcare professionals. A systematic review and meta-synthesis of qualitative studies
Source: Disabil Rehabil. 2024 Aug 5;47(7):1644–58. doi: 10.1080/09638288.2024.2384624 (PMC11974919; doi:10.1080/09638288.2024.2384624)
Supplement: Supplemental Material [file IDRE_A_2384624_SM8107.zip › Supplementary file 2 secondary analysis.docx]

**Supplementary file 1:**

Results of the secondary analysis: number of studies, per country, represented within the themes.

Total number of countries within this theme: 14

Total number of articles within this theme: 49

Total number of countries within this theme: 12

Total number of articles within this theme: 33

Total number of countries within this theme: 9

Total number of articles within this theme: 26

Total number of countries within this theme: 11

Total number of articles within this theme: 36
